# Chemical and physical variations of cannabis smoke from a variety of cannabis samples in New Zealand

**DOI:** 10.1080/20961790.2018.1445937

**Published:** 2018-04-12

**Authors:** Thomas J. Sheehan, Hilary J. Hamnett, Richard Beasley, Paul S. Fitzmaurice

**Affiliations:** a Toxicology, Institute of Environmental Science and Research (ESR), Porirua, New Zealand; b Medical Research Institute of New Zealand, Wellington, New Zealand

**Keywords:** Forensic science, forensic toxicology, cannabis, smoke, delta-9-tetrahydrocannabinol, terpenoids, gas chromatography/mass spectrometry, total particulate matter

## Abstract

Studies have compared the chemical properties of tobacco smoke to those of cannabis smoke, with the objective of identifying the chemical attributes responsible for the mutagenicity and carcinogenicity of cannabis smoke. Comparative studies have included small sample sizes and produced conflicting results. The aim of this study was to assess the major chemical and physical variations of cannabis smoke across a range of cannabis samples of different potencies and origins, sourced from the illegal market in New Zealand. Twelve cannabis samples were studied ranging from 1.0% to 13.4% delta-9-tetrahydrocannabinol (Δ^9^THC) content. A smoking machine was used to smoke “joints” (cannabis cigarettes) and the chemical/physical properties of the smoke assessed. The chemical constituents of the smoke extracts were analysed by gas chromatography/mass spectrometry. A range of different chemical constituents (in addition to Δ^9^THC) were identified and their concentrations estimated. Terpenoids were identified as the major variable in cannabis smoke, showing a 40-fold range in total terpenoid content. Analysis of the total particulate matter showed that significantly different levels of particulate matter were produced between the different cannabis samples, ranging from 14.6 to 66.3 mg/g of cannabis smoked. The Δ^9^THC delivery efficiency during smoking was also investigated and produced consistent results showing a mean and median of 12.6% and 10.8%, respectively, of the theoretically available Δ^9^THC (ranging from 7.2% to 28.0%).

## Introduction

*Cannabis sativa* L. (herein referred to as “cannabis”) is a plant that has a long international history of therapeutic and recreational application [1]. One major concern of cannabis use as a medicine is that the most common route of administration is inhalation via smoking. Literature available on the smoke chemistry of tobacco, leaves little doubt that smoking any plant material produces a range of compounds that have toxic effects on the human body, in particular, carcinogens [2]. Negative health consequences of smoke carcinogens raise questions about smoking cannabis for medicinal purposes.

Like tobacco, the smoking of cannabis has been associated with a diverse range of cancers [3,4]. Work conducted in North Africa found an association between lung cancer and cannabis smoking [5–7], whereas USA case-control [8] and retrospective cohort [9] studies failed to find an association between cannabis smoke and lung cancer. These studies may have been influenced by confounding variables such as the combining of cannabis and tobacco in cannabis joints, or the fact that cannabis smokers are often also tobacco smokers [10]. Furthermore, a large pooled analysis of published and unpublished data found little evidence to suggest an increased risk in lung cancer [11]. A case-control study undertaken in New Zealand (NZ), where the use of tobacco/cannabis mixtures is less common, showed an 8% increase in the risk of lung cancer for a cannabis smoker per joint year (one “joint year” being the equivalent of one joint per day for one year) [10].

Research has focused on the effects that smoking cannabis has on the function of human lungs, as it is reasonable to assume that they will be similar to that of tobacco smoke, which is known to lead to conditions such as chronic obstructive pulmonary disease [12]. It has been suggested that the effects of one joint are equivalent to that of 2.5–5 tobacco cigarettes in terms of airflow obstruction and hyperinflation, due to the way cannabis is smoked, i.e. without a filter, with a shorter butt length, and with deeper/longer inhalation [13].

A review of molecular biological techniques to assess the toxicity of cannabis smoke showed contradictory results. These studies also used a limited variety of cannabis samples [14–16]. Maertens et al. [16] noted that although their results suggested a higher level of toxicity in cannabis smoke than tobacco smoke, it would be necessary to experiment across a variety of cannabis samples.

To date, studies have investigated the composition and properties of cannabis smoke [17–21], and have compared it to cigarette smoke [22–27]. Studies have also examined the composition and properties of cannabis smoke produced by different methods of smoking [28–34]. Most studies have been limited to the comparison of one or two cannabis materials and tobacco products. When two or more cannabis varieties have been compared, only limited quantitative and qualitative differences have been demonstrated [35]. The results are also contradictory. For example, some studies have reported benzo[*a*]pyrene (B[*a*]P) at greater levels in cannabis smoke than tobacco smoke [22], whilst others report cannabis smoke to have less B[*a*]P [27]. A reason for disagreement between the studies of cannabis smoke may be the source or variety of the cannabis being tested, just as different tobacco products show different smoke properties [36]. Cannabis smoke studies have provided substantial information on the constituents of cannabis smoke, however it is difficult to draw any conclusions regarding the variability from different sources of cannabis.

Most research investigating cannabis smoke has used a single source of cannabis [23,27], and in studies where more than one cannabis sample was used, the sample size and range were limited with respect to relative potency (e.g. two cannabis samples with potencies of 1.3% and 4.5% delta-9-tetrahydrocannabinol (Δ^9^THC) [25], or 1.3% and 4.4% Δ^9^THC [26]). Other than the single study by Fischedick et al. [33], using medicinal cannabis from a single source (6.2%, 10.3% and 21.7% Δ^9^THC), the highest plant Δ^9^THC content previously tested was 4.5% [25] and the lowest was 0.3% [35]. A study by Sparacino et al. [26] states that, despite knowledge of higher Δ^9^THC levels, the cannabis sample potencies tested were a practical representation of the market.

With contradicting conclusions and results on the use, toxicity and constituents of cannabis smoke, we chose to investigate the composition of cannabis smoke from a larger sample set and greater variety of cannabis samples. It may be that some strains, potencies or growing conditions of cannabis contribute variability to smoke constituents, and the delivery of active constituents to the smoker. The primary aim of this study was to compare the differences in cannabis smoke composition when a range of cannabis samples were analysed in the same laboratory, under the same conditions, using the same techniques. The present study fills in the gaps of the analysis of cannabis smoke from a broader range of cannabis potencies and cannabis samples available in NZ, from the illicit street market from different sources and growing conditions, as opposed to cannabis grown under strict pharmaceutical conditions or smoked under different conditions.

## Materials and methods

### Smoking machine

**Figure 1.**
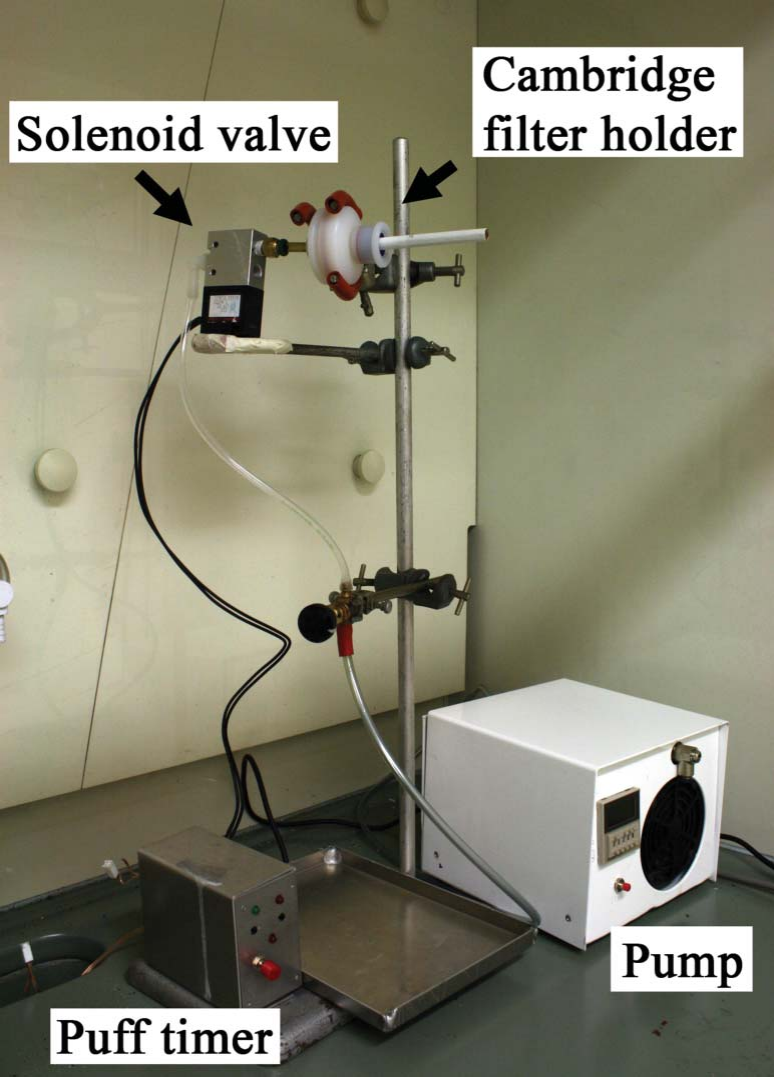
The smoking machine used in this study.

A smoking machine was used, which was made in-house with parts adapted from a TE-2 smoking machine, purchased from Teague enterprises (Woodland, USA), and parts purchased from Cerulean (Milton Keynes, UK) to meet as many criteria as possible of the International Organization for Standardization (ISO) standard for routine analytical cigarette-smoking machines [37]. Smoking experiments, performed under the ISO conditions of 35 mL puffs with a duration of 2 s at intervals of 60 s, used Kentucky reference cigarettes (3R4F) as controls. We measured the total particulate matter (TPM) weights and nicotine levels in the TPM in order to validate the in-house smoking machine and repeatability of nicotine quantitation using the gas chromatography/mass spectrometry (GC/MS) methods described [38]. The repeatability of nicotine quantitation and the expected amount of TPM agreed with the expected variability from smoking machines found in international studies [39]. Figure 1 shows the smoking machine used in this study.

Cannabis samples were ground in a SharpStone® grinder specifically designed for the task of grinding cannabis, and purchased from *Cosmic Corner* in Wellington, NZ. We inserted approximately 650 mg of each ground cannabis sample into Gizeh Silver tip King Size Cigarette tubes™ using an Aztec Ezyfil Tube filling machine™ purchased from *Gentlemen's Corner*, Auckland, NZ. Following preliminary experiments, the mass of 650 mg of cannabis was chosen as the most consistent that could be packed into the cigarette tubes used. To ensure smoking consistency, each joint was stopped after the puff that would leave the butt length as close as possible to 20 mm.

### Chemicals and standards

Certified Δ^9^THC (1.000 ± 0.043) mg/mL and nicotine (1.000 ± 0.006) mg/mL reference standards in MeOH were purchased from Cerilliant® Analytical Reference Standards (Round Rock, USA). Isopropyl alcohol (IPA) and MeOH (analytical grade) were purchased from Thermo Fisher (North Shore City, NZ). The Cambridge filter holder (used to collect TPM from the cigarette smoke) was purchased from Cerulean.

### Cannabis plant samples

Cannabis plant samples (a total of 12) acquired from the Institute of Environmental Science and Research (ESR), NZ came from police cannabis seizures in the North Island of NZ. We selected cannabis samples, using limited information, to reflect different areas of NZ, different growing conditions and different types of plant material (full sample information cannot be provided due to legal restrictions).

### Δ^9^THC Content of cannabis plant samples

The Δ^9^THC content of the cannabis plant samples was measured by air-drying a sub-sample overnight in the laboratory before accurately weighing 100 mg into 10 mL test tubes [40]. The samples were extracted by sonication for 15 min in 5 mL of IPA. The solvent was transferred to a 50 mL volumetric flask via a 0.45 µm nylon syringe filter. This extraction was repeated three times and the extracts added to the same volumetric flask. The sample tubes were washed three more times and combined with the previous extracts, and the 50 mL flasks were made up to volume with IPA. Semi-quantitation of Δ^9^THC content by GC/MS used the parameters listed in Table 1 [41,42]. The Δ^9^THC content of the cannabis plant material is hereafter referred to as *C*_THC_. Preliminary experiments with Δ^9^THC extraction showed that the procedure produced consistent results for *C*_THC_ from the same cannabis sample (when extracted in triplicate). Given limited sample sizes, we tested each cannabis sample for *C*_THC_ once from a sub-sample that represented the cannabis smoked in the smoking experiments.

**Table 1.** Gas chromatography/mass spectrometry (GC/MS) parameters for the analysis of Δ^9^THC.

Equipment/parameter	Specification
GC/MS	Shimadzu QP 2010 GC/MS fitted with a PAL AOC5000 autosampler
Analytical column	Thermo Scientific TraceGOLD TG-5MS, 30 m × 0.32 mm, 0.25 µm, 5% diphenyl/95% dimethyl polysiloxane
Injection port temperature	250 °C
Injection port liner	Restek Custom Liner 3.5 mm × 5 mm, 95 mm, with glass wool
Injection volume	1 µL
Injection mode	10:1 split
Column flow-rate	1.32 mL/min
Transfer line temperature	260 °C
Ion source temperature	250 °C
Data acquisition	Scan *m*/*z* = 35–500
Ion used for semi-quantitation	*m*/*z* = 299
Reference ions	*m*/*z* = 231 and 314
Temperature programme	200 °C for 2 min 10 °C/min to 240 °C 240 °C for 15 min

A semi-quantitation using the Δ^9^THC standard diluted in IPA to concentrations of 50, 100 and 200 µg/mL was performed. Samples were diluted with IPA when necessary to obtain a result inside the calibration curve.

The cannabis Δ^9^THC extract concentration from a sample requiring a two-fold dilution is used in the following example to demonstrate how we calculated the *C*_THC_ of the plant material:

(1) Total Δ9THC collected mg=Conc. µg/mL1 000 µg/mg×50 mL (total volume)×dilution=145.5 1 000×50×2=14.6 mg.

(2) $$\begin{array}{l} C_{\mathrm{THC}}\\ =\frac{\mathrm{Total}\;\Delta^{9}\mathrm{THC collected}(\mathrm{mg})}{\mathrm{Weight of cannabis extracted}(\mathrm{mg})}\times 100\\ =\frac{14.6}{108.8}\times 100\\ =13.4\mathrm{\%.} \end{array}$$

### Joint smoking parameters, extraction and analysis of TPM

Joints were analysed in batches of five after pre-conditioning for a minimum of 48 h at 25 °C and 60% relative humidity. Before smoking, we cut the cigarette filter from the end to represent smoking without a filter [10]. During smoking, the TPM of the mainstream smoke (MSS) of all five joints was collected on a single pre-weighed Cambridge filter holder (containing a filter). The holder and joint butts of the joints were re-weighed to determine the mass of the TPM and the total amount of cannabis smoked. Because of the inability to stop a joint being smoked at an exact butt length or after a consistent number of puffs, we recorded the total mass of cannabis smoked in each session and calculated the TPM per gram of cannabis smoked to normalize the amount of TPM. After removing the “TPM” filter, a second filter was used to wipe any remaining TPM from the inside [43]. Both filters were placed in a 50 mL conical flask and 20 mL of MeOH added, before shaking at 200 rpm for 30 min, filtering using 0.45 µm polyvinylidene difluoride syringe filters and transferring to 2 mL amber vials for GC/MS analysis using the conditions listed in Table 2.

**Table 2.** Gas chromatography/mass spectrometry (GC/MS) parameters for the analysis of cannabis smoke extracts.

Equipment/parameter	Specification
GC/MS	Shimadzu QP 2010 GC/MS fitted with a PAL AOC5000 autosampler
Analytical column	Thermo Scientific TraceGOLD TG-5MS, 30 m × 0.32 mm, 0.25 µm, 5% diphenyl/95% dimethyl polysiloxane
Injection port temperature	260 °C
Injection port liner	Sigma Focus split/splitless liner, 3.4 mm ID with glass wool
Injection volume	1 µL
Injection mode	Splitless injection
Column flow-rate	2.00 mL/min
Transfer line temperature	260 °C
Ion source temperature	260 °C
Data acquisition	Scan *m*/*z* = 41–500
Temperature programme	50 °C for 5 min 3 °C/min to 260 °C 260 °C for 15 min

Compounds in the methanolic extraction of the TPM were identified by comparison with a Wiley MS library (7th edition, 80% spectral match). We used a response factor from an external nicotine standard curve, run at concentrations of 3.1, 12.5 and 25.0 µg/mL (*R*^2^ ≥ 0.98), to estimate the concentration of compounds, other than Δ^9^THC, identified in the smoke extracts [36]. Interpretation of results for compounds other than nicotine and Δ^9^THC should therefore be considered semi-quantitative and for comparative purposes between the samples of cannabis. Nicotine was not found in the cannabis samples.

As we found Δ^9^THC at high concentrations in all the TPM extracts, we determined the Δ^9^THC concentration using appropriate dilutions. We analysed each cannabis sample in duplicate and assessed the variability using the appropriate statistics [44].

To estimate the concentrations of the different chemical constituents in the cannabis smoke extracts, we selected 3 cannabis samples (1.0%, 13.4% and 9.0% *C*_THC_) from the 12 samples and analysed them in duplicate to determine identifiable compounds. We integrated peaks in the total ion chromatograms (TICs) with parameters set to a minimum total ion current peak height of 200 000. We chose this peak height to represent approximately five times the signal-to-noise ratio. Limitations of the MS library meant that the identification of the cannabinoids eluting close to Δ^9^THC could not be completed, and is therefore excluded. This method was used as a pragmatic way of screening, given the number of different compounds/standards available and the unknown variation.

### Delivery efficiency of Δ^9^THC in cannabis smoke

The Δ^9^THC concentrations measured in the TPM allowed us to calculate the total amount of Δ^9^THC delivered in the joint-smoking process, referred to as the “delivery efficiency”. The delivery efficiency is calculated by determining the total amount of Δ^9^THC in the TPM and dividing this by the amount of Δ^9^THC theoretically available in the mass of cannabis smoked:

(3)  Δ9THC delivery efficiency (%) = Total Δ9THC in TPM (mg)Mass smoked mg ×CTHC100×100.

Measuring the Δ^9^THC delivery efficiency is important because previous studies have suggested that most of the Δ^9^THC in the joint does not transfer to the MSS [32,45], whilst others have reported that over 60% is transferred to the MSS under different combustion conditions [30,46].

### TPM Accounted for by Δ^9^THC

We also determined the percentage of TPM, for all duplicate results, accounted for by Δ^9^THC, i.e. the percentage of the TPM by mass that was Δ^9^THC:

(4) Percentage of TPM accounted for by Δ9THC = Total Δ9THC in TPM (mg)TPM (mg)×100.

Determining the percentage of TPM that is accounted for by Δ^9^THC is important because it may provide information regarding harm reduction, i.e. cannabis materials that release higher levels of Δ^9^THC in smoke relative to other particulates. Similar studies have investigated this idea using vaporizers [29].

### Statistical analysis

Analysis of variance (ANOVA) and the *t*-test were used in this study to determine if the variables measured were statistically different between cannabis samples. ANOVA is a statistical method commonly used to determine if there are significant differences between the sample means of data-sets. To test for significant differences between only two means, the *t-*test can be performed. Using either test, results are generally considered significant if the *P*-value < 0.05 (95% probability) and this was deemed appropriate for the results in this study [47]. Statistical tests were performed using Microsoft Excel 2010, version: 14.07194.5000.

## Results

### Cannabis sample Δ^9^THC content

There was a range (1.0%–13.4%) in *C*_THC_ in the 12 cannabis samples, as shown in Table 3. One sample, which consisted mainly of cannabis leaf material (as opposed to flowering heads), had the third lowest level of *C*_THC_ (1.9%). Whilst we had some information regarding the geographical location of the cannabis, our sample numbers prohibit meaningful regional comparisons.

**Table 3.** The Δ^9^THC content (*C*_THC_), total particulate matter (TPM), delivery efficiency and mass of terpenoid data for the cannabis samples in this study.

*C*_THC_ (%)	TPM per gram of cannabis smoked (mg/g) (Rep 1)^a^	TPM per gram of cannabis smoked (mg/g) (Rep 2)^a^	Percentage of TPM accounted for by Δ^9^THC (%) (Rep 1)^a^	Percentage of TPM accounted for by Δ^9^THC (%) (Rep 2)^a^	Δ^9^ THC delivery efficiency (%) (Rep 1)^a^	Δ^9^ THC delivery efficiency (%) (Rep 2)^a^	Total mass of terpenoids per gram of cannabis smoked (µg/g) (Rep 1)^a^	Total mass of terpenoids per gram of cannabis smoked (µg/g) (Rep 2)^a^
1.0	40.2	44.2	3.6	4.1	14.6	18.6	200	300
1.4	14.6	17.7	9.7	9.8	10.5	12.9	50	70
1.9	17.2	16.0	15.4	10.6	13.7	8.7	80	90
2.7	25.6	40.2	10.8	6.7	10.2	9.9	1000	1400
5.8	37.1	58.5	32.8	27.9	20.9	28.0	1100	1500
9.0	41.1	34.1	21.2	26.7	9.7	10.2	1200	1100
9.1	47.9	40.0	31.8	32.0	16.7	14.1	2300	2200
10.5	46.9	54.0	18.9	24.3	8.5	12.6	1400	2100
10.9	45.5	66.3	31.1	20.5	13.0	12.4	2700	2600
11.0	41.2	29.0	23.1	28.4	8.3	7.2	2100	1400
12.2	61.0	58.1	20.6	20.4	10.4	9.7	4000	4300
13.4	61.0	63.8	24.8	22.8	11.0	10.6	2600	1900

a Results from duplicate experiments of the cannabis smoke TPM collected from five joints on a single Cambridge filter. Rep 1 and rep 2 refer to measurements of TPM taken from the same cannabis sample during different smoking sessions on different days, each time collected from five joints. Values below 1 000 rounded to 1 significant figure and values above 1 000 rounded to 2 significant figures.

### TPM Collected from joints

The mass of TPM produced from the cannabis samples ranged from 14.6 to 66.3 mg/g (Table 3). We observed, using ANOVA of the duplicate results in Table 3, significant differences in the levels of TPM per gram of cannabis smoked between the different cannabis samples (*P* < 0.001), although there was no clear relationship to a factor other than the samples being different sources of cannabis.

### Δ^9^THC Levels in the cannabis smoke TPM

The percentage of TPM accounted for by Δ^9^THC appears to separate into two groups; four cannabis samples with *C*_THC_ < 5.8% produced one group where the Δ^9^THC accounts for a lower percentage of the TPM (mean 8.8%), and the higher *C*_THC_ samples (*C*_THC_ ≥ 5.8%) produced TPM where the Δ^9^THC accounts for a much higher percentage (mean 25.5%) (see Equation (4)). The groups were treated as independent means and a two-tailed unpaired *t*-test was performed showing a strong significant difference between the two: *t*_stat_ = −9.14, *t*_crit_ = 2.11, *P* < 1 × 10^−^^7^ (see Table 3 for individual sample results).

The delivery efficiency of Δ^9^THC during smoking is reasonably consistent across all the cannabis samples (Table 3). The mean and median delivery efficiencies are 12.6% and 10.8%, respectively (range 7.2–28.0%). The cannabis sample with *C*_THC_ = 5.8% differs from the other samples, delivering a higher percentage of the theoretically available Δ^9^THC. The 5.8% *C*_THC_ sample is also isolated from the other cannabis samples in terms of potency, with the nearest potencies being 2.7% and 9.0% *C*_THC_ [38]. The numerical results are listed in Table 3.

### Compounds detected in the cannabis smoke TPM

**Figure 2.**
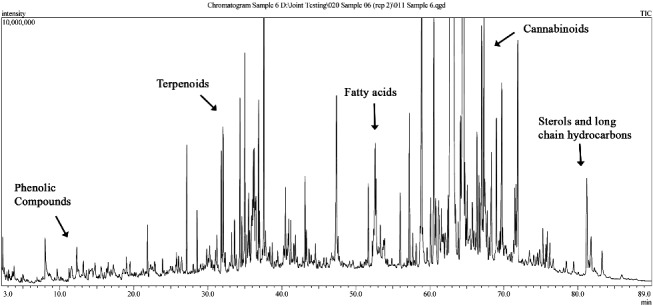
An example of a total ion chromatogram (TIC) of the total particulate matter (TPM) collected from five cannabis joints of a single cannabis sample (12.2% *C*_THC_).

As expected, we found the most abundant groups of compounds in the cannabis smoke TPM to be cannabinoids. Figure 2 is an example TIC of a cannabis smoke TPM extract with labels indicating the areas of cannabinoids and other classes of identifiable compounds. We examined all of the cannabis smoke extract chromatograms, with three chosen for thorough interpretation (1.0%, 13.4% and 9.0% *C*_THC_) to cover the range of Δ^9^THC potencies observed.

### Terpenoids

We investigated all 12 cannabis samples further for terpenoid content. The region in the chromatograms associated with terpenoids showed a general increase in both number and total concentration of terpenoids detected with increasing *C*_THC_. ANOVA of the results showed that the number of measurable terpenoids in the lowest *C*_THC_ sample is significantly lower than the number in samples with higher *C*_THC_ (*P* < 0.001). In the three lowest *C*_THC_ cannabis samples (1.0%, 1.4% and 1.9% *C*_THC_), only three or fewer terpenoids were measureable. At higher *C*_THC_ levels (12.2% *C*_THC_), we detected 25 different terpenoids and estimated their yields. The total mass of terpenoids (µg) per gram of cannabis smoked can be used as an indicator of the total variability of terpenoid content in different cannabis samples (Table 3).

**Figure 3.**
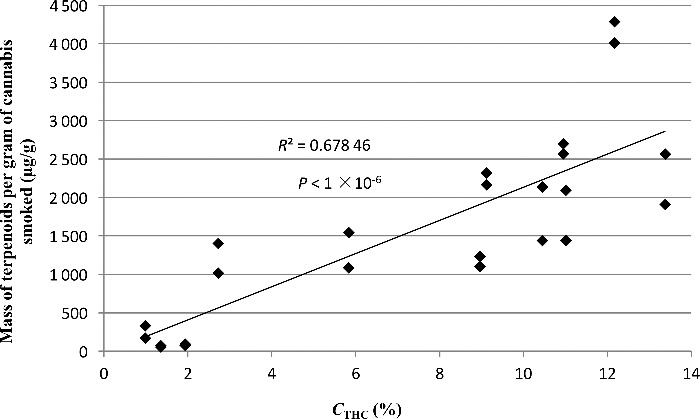
Scatter diagram of the estimated total mass of terpenoids in the TPM *vs*. the Δ^9^THC content (*C*_THC_).

The variability observed in the total mass of terpenoids per gram of cannabis smoked *vs*. *C*_THC_ may be a natural variable of the smoking process. The results strongly indicate a general increase with increasing *C*_THC_ (Figure 3 and Table 3). Variability in the identity of the different terpenoids from each cannabis TPM extract can also be seen. Table 4 shows the terpenoids identified in all 12 samples. Some terpenoids in Table 4 are listed multiple times due to the lack of readily available standards, and ambiguity in the identification of terpenoids in cannabis as there are more than 20 000 known terpenoids [48]. However, based on the order of elution, retention times (RTs) and library matching, any repeats of minor isomers are distinguishable as different terpenoids.

**Table 4.** Terpenoids identified and their estimated mass in the cannabis smoke TPM extracts, presented as µg/g of cannabis smoked (Rep 1, Rep 2).

	*C*_THC_ (%)											
Terpenoid	1.0	1.4	1.9	2.7	5.8	9.0	9.1	10.5	10.9	11.0	12.2	13.4
*trans*(β)-Caryophyllene	30, 50	NQ	NQ	290, 340	90, 150	200, 190	260, 230	210, 200	290, 300	230, 150	190, 210	130, 100
α-Bergamotene	–	NQ	NQ	30, 40	50, 80	60, 50	60, 50	80, 80	160, 150	180, 110	30, 30	70, 50
α-Humulene	–	NQ	NQ	90, 120	40, 60	80, 80	100, 80	80, 110	120, 120	90, 60	100, 100	80, 50
β-Farnesene	–	–	–	NQ	–	–	–	NQ	90, 90	60, 40	–	50, 80
β-Selinene	–	–	–	–	NQ	–	–	–	80, 80	90, 60	60, 80	–
α-Selinene	–	–	–	70, 50	30, 70	70, 70	50, 40	–	130, 110	80, 50	100, 110	50, 70
δ-Guaiene	–	–	–	–	–	90, 90	70, 70	90, 90	260, 260	310, 200	–	–
Guiana-3,9-diene	–	–	–	30, 50	–	–	40, 40	40, 50	–	–	–	–
β-Bisabolene	–	–	–	–	–	NQ	–	–	50, 50	40, 70	60, 60	–
Neoalloocimene	–	–	–	–	–	–	–	–	110, 100	60, 20	80, 80	–
Valencene	–	NQ	NQ	70, 100	70, 110	110, 100	130, 110	110, 110	240, 230	200, 130	180, 190	110, 80
Selina-3,7(11)-diene	–	NQ	30, 30	110, 150	110, 160	160, 140	190, 160	160, 180	350, 330	300, 200	250, 270	190, 140
α-Bisabolene	–	–	NQ	–	–	–	–	–	30, 30	40, 30	40, 40	130, 100
Nerolidol B (*cis* or *trans*)	NQ	–	–	–	50, 70	70, 60	70, 60	50, 90	120, 80	100, 70	80, 90	110, 70
(–)-Caryophyllene oxide	–	–	–	130, 220	30, 60	70, 70	70, 80	50, 120	100, 90	40, 30	120, 140	80, 60
Guaiol	–	–	NQ	–	90, 150	60, 50	200, 200	50, 100	–	–	290, 320	240, 180
(–)-Caryophyllene oxide	90, 170	–	–	40, 60	30, 40	–	50, 50	30, 70	40, 40	–	90, 100	–
10-epi-δ-Eudesmol	–	–	NQ	30, 50	90, 50	50, 50	210, 210	50, 100	–	–	360, 390	230, 170
Veridiflorol	–	–	–	–	–	–	100, 30	40, 90	–	–	190, 200	–
(+)-Aromadendrene	NQ	–	–	30, 60	NQ	NQ	–	–	40, 30	–	70, 80	50, 30
Elemol	–	–	–	–	–	–	90, 60	40, 70	–	–	90, 90	120, 50
β-Eudesmol	–	–	NQ	–	80, 100	–	130, 140	30, 80	–	–	200, 200	150, 110
α-Eudesmol	–	–	NQ	–	90, 120	–	150, 160	70, 140	–	–	220, 230	170, 130
Epiglobulol	–	–	–	–	–	–	–	–	–	120, 90	–	–
Palustrol	–	–	–	–	–	–	–	–	–	–	120, 130	–
*trans-*Longipinocarveol	–	–	–	–	–	–	–	–	–	–	120, 120	–
Bulnesol	–	–	30, 30	–	100, 160	50, 40	180, 190	40, 100	–	–	260, 280	230, 180
(–)-Caryophyllene oxide	50, 110	NQ	NQ	60, 90	–	50, 40	40, 60	40, 100	150, 150	–	110, 120	70, 70
α-Bisabolol	NQ	50, 70	30, 30	40, 70	130, 180	100, 90	140, 150	170, 260	330, 310	180, 140	570, 620	290, 190

No entry in the table means that the terpenoid was not detected; NQ: the terpenoid was detected but its concentration could not be estimated. Any compounds repeated in the table are based on the best matching mass spectrum in the MS library, however, based on the retention times (RTs) it can be confirmed that they are all different terpenoids. Results are rounded to the nearest 10 µg/g; rep 1 and rep 2 refer to measurements from the same cannabis sample during different smoking sessions on different days, each time collected from five joints.

## Discussion

The analysis of a range of cannabis samples has identified novel properties of cannabis smoke, which may be linked to the source or type of cannabis material.

The cannabis *C*_THC_ levels obtained indicate that the cannabis samples used cover a realistic range, based on national and international work [40–42,49–53]. It has often been noted in international studies that over time, there has been an increase in the *C*_THC_ of cannabis. A published NZ study, analysing cannabis from the illegal market, reported cannabis *C*_THC_ levels between 1976 and 1996 [40], and indicated that at the time of publication (2000), no cannabis above 10% *C*_THC_ had been tested in NZ. More recent testing in NZ has shown a maximum of 18.1% *C*_THC_ cannabis (Personal communication, Robyn Somerville ESR, 2016). This study contains five samples that are above 10%, with a maximum of 13.4% *C*_THC_ and may indicate an increasing trend in cannabis *C*_THC_ levels on the NZ market. A grow study performed in NZ showed that cannabis sourced in NZ has the potential to produce flowering heads containing up to 30% *C*_THC_ [54], although this has yet to be seen.

Our results indicate significant differences in the levels of TPM received by a smoker from different cannabis samples. There is some suggestion from our results of a general increase in TPM with increasing *C*_THC_; however, exceptions, notably, that the lowest *C*_THC_ material (1.0%) produced a significantly higher level of TPM when compared with the two closest *C*_THC_ samples (1.4% and 1.9%). A possibility for this observation may be the growing conditions of the cannabis samples, or the type of plant material.

The percentage of TPM accounted for by Δ^9^THC correlated to *C*_THC_, shows that the smoker is receiving more Δ^9^THC per unit of TPM when smoking cannabis material with a higher *C*_THC_. This is expected, as the higher *C*_THC_ accounts for an increased proportion of TPM in the smoke.

As mentioned above, the Δ^9^THC delivery efficiencies are reasonably consistent across all the cannabis samples, but it is not clear why the 5.8% *C*_THC_ cannabis sample is an outlier (although not statistically so). A study by Fehr and Kalant [46] reported higher Δ^9^THC delivery efficiencies (between 34.2% and 62.2%) however, they used different flow-rates/puffing conditions for a single cannabis sample, rather than constant smoking behaviour for multiple cannabis samples. Fehr and Kalant also combusted the entire cannabis material in the joint (including the butt) and thus their results are not directly comparable with ours. The results reported here represent, in our view, realistic delivery efficiencies under the smoking conditions used. Estimations that up to 50% of the Δ^9^THC is lost in sidestream smoke, up to 30% is destroyed by pyrolysis and 10% is trapped in the butt of the joint have been reported [32,45]. The present study fills in the gaps of accessing Δ^9^THC recovery from multiple cannabis samples with a diverse range of potencies. Our results indicate that the percentage of available Δ^9^THC delivered in the MSS remains consistent when the joints are smoked in a consistent way. When the Δ^9^THC delivery efficiencies and percentages of TPM accounted for by Δ^9^THC are considered together, it suggests that the combustion and composition of cannabis smoke are not consistent between samples from varying sources.

There have been suggestions that terpenoids play an important role in the therapeutic effects of cannabis [33,55,56]. Some research promotes the concept that cannabis has other herbal and synergistic components, as well as Δ^9^THC, and therefore medicinal products containing only Δ^9^THC are potentially less effective therapeutically [56]. Various terpenoids that are found in cannabis are known to have pharmacological properties themselves. For example, caryophyllene (detected in this research) is reported to be an agonist to the CB_2_ receptor and to provide anti-inflammatory effects [57]. β-Elemene (also detected) has been shown to have anti-carcinogenic properties [58]. In contrast to the potential therapeutic applications of terpenoids, there is also evidence that they are precursors for poly-aromatic hydrocarbons [59], which raises the question of toxic side-effects. The results of the terpenoids analysis in this study show that not only is the total mass of terpenoids greater in cannabis samples with higher *C*_THC_ levels, but there is a significant variability in the terpenoids in each sample. Previous research showed similar correlations between terpenoid and cannabinoid composition in cannabis plant material [55], and there are also reports of terpenoids as a major constituent of cannabis smoke [33]. In contrast, our results suggest that terpenoids are one of the most variable constituents of cannabis smoke with potentially a 40-fold variation between samples. Whilst the study by Fischedick et al. [33] identified terpenoids as a major constituent of cannabis smoke, it was not directed at the variability of terpenoids, but rather the binding affinity of smoke extracts to cannabinoid receptors. Hence, Fischedick et al. [33] may not have identified the variability of terpenoids presented here.

The terpenoids identified in this study are primarily sesquiterpenoids, but monoterpenoids are also present. This could be due to the taxonomy of the cannabis samples grown in NZ or a consequence of cannabis storage. It has been shown that the composition of terpenoids in the production of essential oils not only depends on the method of production of the oil but also on growing conditions such as soil, climate, growth stage, harvest time and previous storage of the plant material [60].

## Conclusion

In conjunction with cannabinoids, we found terpenoids to be the most variable component of cannabis smoke. Our results show that in cannabis smoke, terpenoids show a total increase with increasing Δ^9^THC content. The potential for terpenoids to be precursors for carcinogens highlights the question of smoking technique, as smoking cannabis joints with higher levels of Δ^9^THC may lead to higher levels of carcinogens such as B[*a*]P.

There is a general increase in the TPM of cannabis smoke with increasing cannabis plant Δ^9^THC content. An increased level of TPM may have detrimental effects on the user, including carcinogenicity and respiratory irritation, and the Δ^9^THC results from this study also show that when cannabis is smoked under the same conditions, the Δ^9^THC delivery efficiency is consistent across a range of cannabis potencies. In terms of delivery efficiency, we found the cannabis sample with a potency of 5.8% *C*_THC_ to be more efficient than the other cannabis samples (approximately 25% delivery efficiency). Other than the single outlier, the mean and median delivery efficiencies of Δ^9^THC are approximately 13% and 11%, respectively, and the 5.8% *C*_THC_ sample lies in a potency gap between the other samples.

The results also showed that the percentage of TPM accounted for by Δ^9^THC has a tendency to reach a maximum at higher potencies. This suggests that once the cannabis reaches a certain Δ^9^THC level, the smoker receives a consistently higher ratio of Δ^9^THC to TPM, whereas at lower potencies the smoker receives less Δ^9^THC per unit of TPM, and this raises the question of whether or not there are health benefits in smoking cannabis with a higher *C*_THC_.

The cannabis samples tested showed a range of 1.0–13.4% *C*_THC_ content. Not only has this range of cannabis potencies allowed us to uncover trends in the chemistry of cannabis smoke, but it also validates that the findings are relevant to the toxicology of cannabis smoke produced from a representative sample of the cannabis available in national and international markets.
